# Concussion response and recovery in men and women’s rugby union: A reflexive thematic analysis of player interviews

**DOI:** 10.1371/journal.pone.0296646

**Published:** 2024-04-30

**Authors:** Freja J. Petrie, Kelly A. Mackintosh, Chelsea Starbuck, Elisabeth M. P. Williams, Melitta A. McNarry

**Affiliations:** Applied Sports, Technology, Exercise and Medicine, Swansea University, Swansea, Wales, United Kingdom; Erzurum Technical University: Erzurum Teknik Universitesi, TURKEY

## Abstract

Numerous concussion-management protocols have been developed in rugby, though little is known about player’s personal experiences of concussion. Specifically, research typically refers to clinical recovery, with social and psychological sequelae post-concussion gaining little attention. This study aimed to explore the experiences of rugby players in relation to being concussed and recovering from concussion. UK-based rugby players (10 men, 9 women and 1 non-binary person) from school, university, club, military and semi-professional teams took part in semi-structured interviews (36 ± 12 minutes). Interviews were transcribed verbatim, and a reflexive thematic analysis was conducted. Players considered pitch-side healthcare a necessity, though amateur players highlighted the difficulty in consistently accessing this resource. In the absence of medical staff, players were reliant on the goodwill of volunteers, but their response to concussion did not always align with current concussion guidance. Players highlighted that concussion recovery could be socially isolating and that current return-to-play programmes did little to restore lost confidence, resulting in retirement from the game in some instances. Participants expressed a desire for more in-person concussion education and for greater coverage of holistic methods to support their recovery. This study highlights a need for further investigation of the post-concussion social and psychological changes that players may experience during their recovery. Greater focus on information relating to concussion recovery and return-to-contact in education programmes would likely benefit player welfare.

## Introduction

Rugby union (hereafter referred to as rugby) is a full contact field sport played by millions of people across the world [1]. The contact events inherent within the sport act as a ‘unique selling point’ as players enjoy the physical challenge and outlet for controlled aggression [2]. Whilst these contact events therefore represent an attraction of the sport, their propensity to cause concussion has considerable implications for the current and long-term health of players [3, 4].

Concussion is caused by the transfer of acceleration to the brain, either from a direct impact to the head or via a bodily impact [5]. The incidence of concussion is reported to be 8.7, 4.1, 12.6 and 22.2 concussions per 1,000 playing hours for youth, community, elite women’s and elite men’s rugby within the UK, respectively [6–9]. On an individual level, 82% of community and 94% of professional New Zealand-based players reported experiencing a concussion during their rugby playing career [10]. Given that the symptoms of concussion include amnesia, ataxia, behaviour change, and visual disturbances [5], the relative invisibility and transiency of symptom presentation means that concussion identification and management is challenging [11].

At the elite level, concussion management is aided by plentiful resources and the presence of medical professionals at matches. At the amateur level however, there is a relative dearth of pitch-side healthcare [12, 13], meaning that players, match officials, and spectators are relied upon to identify concussion and provide initial support [14, 15]. Consequently, the appropriateness of the concussion response is dependent on the knowledge of those present. Whilst UK Concussion Guidelines for Non-Elite Sport [16] recommend that players seek the advice of pitch-side medics, or call NHS 111 or 999 depending on resources and symptom severity, such behaviours post-concussion are not routinely undertaken by players [17]. If players do not present to a medical professional, up-to-date concussion advice cannot be disseminated, and players may risk further aggravation of the injury through premature return to sport.

Once concussion symptoms have ceased and the mandatory rest periods been completed, both World Rugby and the UK government recommend the following of a return to play (RTP) protocol. The rugby-specific protocol developed by World Rugby gradually reintroduces normal activities and aerobic exercise, followed by non-contact drills, full-contact practice and, finally, competitive game play [18]. Engagement with RTP protocols is limited however, with only 30.9% of United States high-school athletes overall complying with RTP guidelines [12]. Reasons for non-adherence (highlighted in South African adolescent athletes) include peer pressure to return early, an intrinsic motivation to continue playing, and ignorance despite being aware of the potential consequences of poor concussion management [19]. Player well-being during concussion recovery could be further impacted by difficulties returning to pre-concussion sporting or academic attainment levels with the absence of appropriate rehabilitation, compounded by alienation from peers [20, 21]. Psychosocial changes such as these are frequently reported following concussive injury, yet limited guidance is available to improve these outcomes. To inform the development of such guidance, this study investigated the concussion experiences of rugby players of different levels, and their subsequent navigation through the recovery process.

## Methods

Ethical approval was granted by the Swansea University FSE Research Ethics Committee prior to the start of this study (FP_31-03-22b). A total of 20 UK-based rugby players (ten men, nine women and one non-binary person, Table 1) volunteered to take part in online, semi-structured interviews to discuss their experiences of concussion within rugby. Participants were recruited through social media and email networks (between the 17^th^ of May 2022 and the 10^th^ of April 2023) and had played rugby in school, club, military, semi-professional or university teams. All reference to concussion within the findings of this study will be considered self-reported as clinical confirmation of injury status could not be obtained.

**Table 1:** Participant characteristics.

Code	Gender	Current Player	Previously concussed	Playing country	Playing environments	Rugby experience (years)
W1	Woman	Yes	Yes	Scotland/Wales	Academy/University/Club	10
M2	Man	Yes	Yes	Scotland	Club/University	8
W3	Woman	Yes	Yes	England	Club/University	10
M4	Man	Yes	Yes	Wales	Academy/University/Club	15
W5	Woman	Yes	Yes	England	University/Club	10
W6	Woman	Yes	No	England	University	3
M7	Man	No	No	England	School/Club/University	25
M8	Man	No	Yes	Wales	School/University/Club	11
M9	Man	Yes	Yes	England	Club/University/Semi-professional	17
W10	Woman	Yes	Yes	England	Club	1
M11	Man	Yes	Yes	England/Wales	Club/University	17
M12	Man	Yes	Yes	England/Wales	School/Club	13
W13	Woman	Yes	Yes	Ireland/Wales	Academy/University/Club	17
M14	Man	Yes	Yes	England	Club/Military	30
W15	Woman	Yes	Yes	England	Club/International	11
W16	Woman	Yes	No	Wales	Club	1
M17	Man	Yes	Yes	Wales	Club/University	30
NB18	Non-binary	Yes	No	England	Club	1
W19	Woman	Yes	Yes	England/Wales	Club/University	9
M20	Man	Yes	Yes	England/Wales	Club/University	19

To build a rapport, participants were first asked to describe their journey through rugby prior to answering questions relating to their concussion experience. If the participant discussed knowledge relevant to the research question, probing questions were asked to provide a rich dataset (S1 File: Interview guide). Interviews lasted 36 ± 12 minutes and were manually transcribed verbatim. Any identifiable participant data were anonymised during transcription by the interviewer. A reflexive thematic analysis, as described by Braun and Clarke [22], was conducted by the first author (FJP), a white British woman who experienced multiple concussions throughout her university rugby career.

Reflexive thematic analysis allows for new knowledge to be analysed inductively [23]. Following a data-familiarisation process, initial codes were generated to highlight information aligned with the research question. Having reflected upon the appropriateness of this allocation, these codes were aggregated to form themes. The quality of the analysis was verified by a critical friend in accordance with the method devised by Braun and Clarke [24], and adjustments to the naming of themes were made following the feedback received. The thematic map was subsequently adjusted to account for these changes and a report produced. This process was not linear—FJP moved between stages fluidly throughout the data analysis process. The analysis was conducted using NVivo software (Version 21 QRSE International). Given that each player has a different experience of playing rugby and knowledge amongst rugby communities is socially constructed, this study did not consider reality to be singular. Therefore, this study was aligned with the constructivist paradigm [25].

## Results

**Fig 1:**
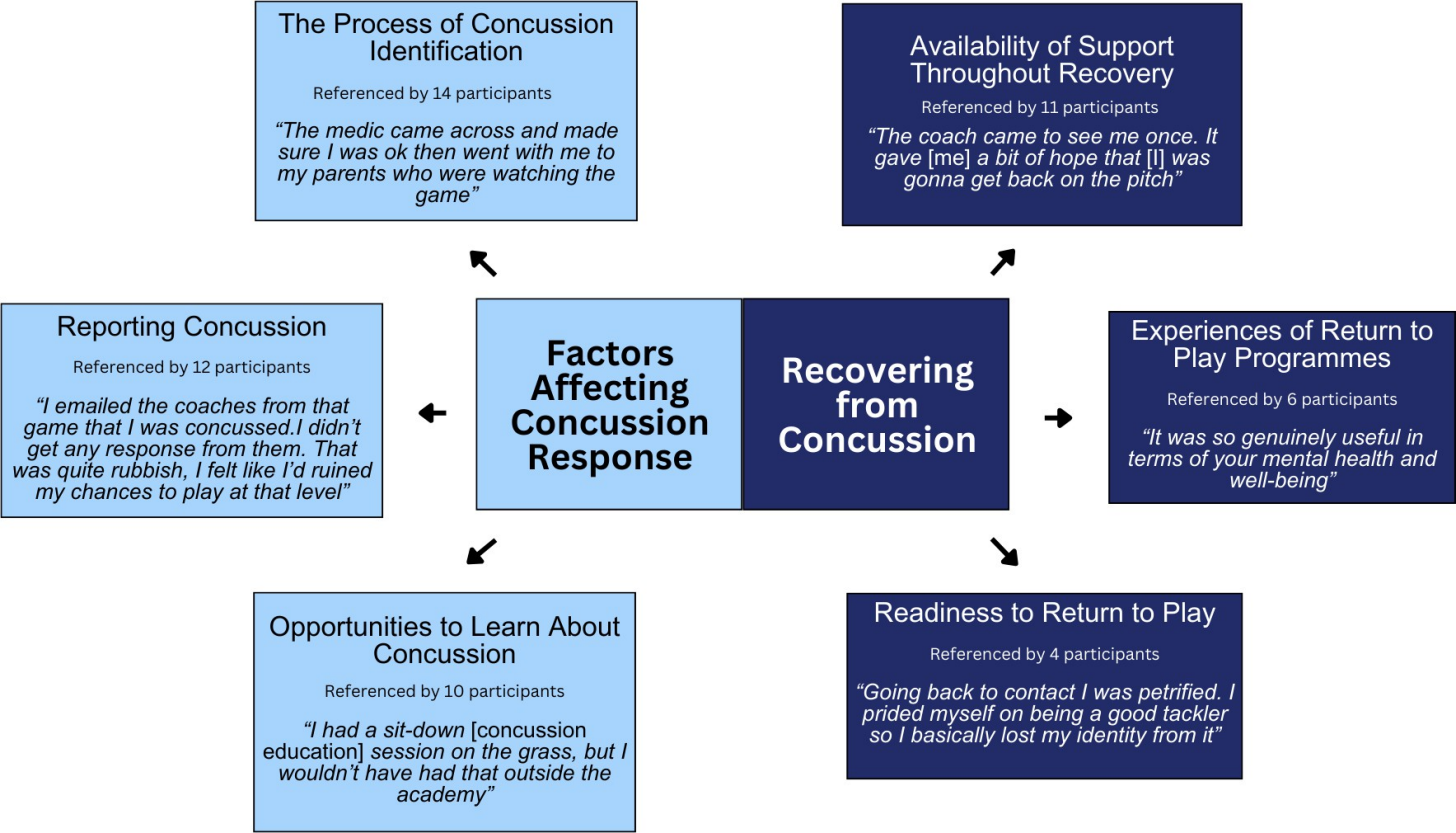
A pen profile indicating the themes and codes identified through reflexive thematic analysis.

Two themes were identified via the analytic process: Factors affecting concussion response and Recovering from concussion (Fig 1). Each theme was associated with three codes. For Factors affecting concussion response, codes were; The process of concussion identification, Reporting concussion and Opportunity to learn about concussion. For Recovering from concussion, codes were; Availability of support throughout recovery, Experiences of return to play programmes and Readiness to return to play.

### Factors affecting concussion response

#### The process of concussion identification

Across genders and playing levels, some concussions were identified *‘immediately’* (NB18) by the referees or pitch-side medical staff, and play was stopped *‘straight away’* (M4). Participants witnessed referees enforcing appropriate concussion protocols despite attempts from players and coaches to dismiss the injury: *‘his coach said*, *oh he’s always like this*, *he’s always making things up*, *but the referee took the boy seriously and treated him absolutely spot on and didn’t dismiss it’* (M11).

W1 described how she suspected she was concussed at a pathway training camp, but that *‘it wasn’t really identified*, *because it was not with people* [she] *knew really well’*. She recalled how *‘only one person really noticed’* but because the camp was a selection opportunity, the player that noticed *‘wasn’t going to argue with the coach’* by suggesting removal from play. Some participants (W1, W19, M11, M17) felt that the coach had a responsibility to identify player concussions: *‘it really annoyed me that she didn’t take me off*, *I went down a couple of times’* (W1). However, whilst this participant expressed that she was aware of her symptoms, she ‘*still didn’t take ownership’* for removing herself from play. On another occasion, W1 reported a concussion to her coach but this reporting was not taken seriously. Later, the coach reconsidered his decision, but angry at his behaviour, W1 defied him and continued playing: *‘I thought well*, *I’m not coming off if you said no*, *it was really stupid*, *I was just being stubborn’*.

Visible symptoms appeared to encourage a more appropriate concussion response (M20, W5). W13 described how she *‘hit’* their head and the referee was going to *‘give them 10 minutes and maybe allow them back on’*, but a lump appeared on her head and the referee then removed her from play. She perceived that ‘*if there wasn’t anything visible*, *he would have said if you feel ok you can go back on*.*’* Some participants recalled being asked basic questions in attempts to identify concussions but noted that the answering of these questions did not accurately indicate their condition (Table 2, Quote 1).

**Table 2:** Supporting quotes for ’Factors affecting concussion response’.

Quote	Code	
1	M4	I managed to overcome an attempt at removing me from play once with a referee. He asked me really basic questions, what day it was, what was the score. But he got the score wrong, I knew the score. But I could see stars, I was like this is ridiculous, I’m taking myself off.
2	W19	A lot of the girls would get hurt and need a few minutes, but you got the feel that they’re not actually hurt, they’re a bit of a drama queen. And I never wanted to be like that, I took pride that I wasn’t one of those.
3	W15	Our physio said if she reports it to [the union] as a serious concussion there are even more protocols to follow. With the insurance for grassroots rugby, if they have two serious concussions the insurance will no longer medically cover them if they get a third. If the player gets a third, they’re no longer insured. They have to fork out the costs so there’s that reluctancy for a club to register a concussion if its low level.
4	W15	If we didn’t have enough front rowers, we would have had to forfeit the game. If you forfeit the game, you get kicked out of the league, we were like she’s gunna have to play. We said do one scrum, go down ‘injured’ and we can do uncontested scrums. We basically fiddled the system, so we didn’t get penalised for not having a front row, but we had to knowingly field an injured player in order to do that.
5	M11	When we played certain teams, their forward pack would dominate us in the scrums. We’d take a prop off at half time because he was (air quotes) injured, then ten minutes into the second half another prop would go down (air quotes) injured. We’d have to [scrum] uncontested and that increased our chances of winning the game. You might have teams that go (air quotes) ooooo concussion.
6	M14	You see the kids coming into rugby trying to copy it. This sort of stuff has to stop. Because it’s not only detrimental to their health, but the other person that they’re running into. It’s dangerous!

Following a concussion, participants were often offered basic advice and were *‘warned’*, or ‘*encouraged’* not to drink (W6), but this advice was not always followed: *‘there’s just the culture of having a drink’* (M2), *‘a lot of times injured players go and get a pint*, [after being removed from play] *they know it’s not good for them to start drinking after a head knock’* (M12). Participants expressed a desire for a post-concussion contract enforced by their club that included *‘instructions to say what you need to do*, *or when you need to see a doctor*,*’* which should be agreed prior to the start of each season. Furthermore, participants (W16, NB18, W3) suggested that teams travelling to matches via bus should assign responsibility to care for injured players and facilitate hospital transport to a non-playing person.

#### Reporting concussion

Reasons for non-reporting of concussion included a desire to be seen as *‘brave’* (M7, M17, W19), risk of voiding insurance cover (W15), perceived low severity (M9, W13, M20), and loss of playing opportunities (M4, W19). The culture of *‘playing through’* pain was an attitude embedded in rugby as *‘there’s still a stigma of being soft’* (Table 2, Quote 2). This culture, reported by both players and coaches, was reportedly more present in adolescents as older players were more likely to consider their *‘future health*, *not just their current health’* (M17, M4). Additionally, participants were frustrated that concussion reporting resulted in a loss of opportunity to play in higher-level teams. W19 had worked hard to upskill but got concussed the week before her first team debut: *‘I ruined my chances because I was stupid enough to self-identify my concussion’* (W19).

Other participants described how they did not intentionally decide to not report their concussion symptoms, rather that they believed that their symptoms were not concussion related, or perceived them as low severity (M9, M20, M11). Further, participants who experienced facial injuries during concussive events described how these symptoms were more painful and visible, therefore concussion reporting was less likely: *‘I was more worried about my broken nose*, *the symptoms were worse’* (M9). On another occasion, M9 misattributed a post-concussion headache to heatstroke and noted that he did not report these symptoms as he *‘didn’t know much about concussions at the time’* and that it *‘didn’t come up in conversation’* with his parents.

Some instances of non-reporting were to avoid consequences imposed upon players by unions or insurance companies. Participants felt comfortable reporting their concussions to medical professionals but were less keen to report to the rugby unions for fear of voiding their insurance policies (Table 2, Quote 3). For others, concussion reporting was also discouraged if playing numbers were low: *‘he’s a lovely guy*, *but we didn’t have subs that day’* (M2), *‘I was trying to pretend it was fine*, *they* [the opposition] *had a big bench*, *but we would have to go down to 14* [players]*’* (W1), but this experience was not universal: *‘as much as we struggle at grassroots to field a team sometimes*, *I’ve never had that pressure to play on*, *and that’s why I continue to play’* (W6, NB18).

Concussion reporting was also influenced by a desire to avoid the penalties given by unions for teams that were unable to field a team for a fixture (Table 2, Quote 4), with players *‘fiddling’* the system. W15 expressed that this was inevitable as long as the penalties for not fielding a team were so high: *‘you are always going to promote*, *encourage people to play injured or concussed players*.*’* She noted how injured players would be told to *‘stay out of the way’* on the wing during the match, but that this was seldom possible as *‘there are so many external forces at play*.*’* Further, W15 compared the strict penalties for not fielding a team to the lack of respective penalties if teams were affected by the COVID-19 pandemic. The *‘covid card’* (since recalled) allowed teams who had tested positive for COVID-19 to forfeit matches without consequence *‘because it was a pandemic*, *and everyone’s safety was thought about*.*’* W15 expressed frustration at this rule and maintained that forfeits for lack of players were equally detrimental to player welfare and that teams should not be penalised for not playing concussed or injured players. Despite being equally critical of the union-inflicted penalties, M11 acknowledged that *‘there would always be a grey area where some teams try to play on that to win’* (M11) and considered that a blanket *‘concussion card’* may result in ‘*clubs using that to their advantage*.*’* M11 then explained how strategic injury reporting was currently and commonly used by players during games to force the use of uncontested scrums and gain an advantage over a dominant scrummaging pack (Table 2, Quote 5).

#### Opportunities to learn about concussion

Participants did not typically receive formal concussion education. Those that trained in higher-level or pathway environments had received formal education that took the format of medic-led group discussions, lectures, or compulsory e-learning sessions. The efficacy of the e-learning was questioned as players could skim through without taking in the content, however, some (W15, NB18, M20) believed that it was useful to an extent, suggesting that younger players *‘who hadn’t really given it* [concussion] *a thought’* would be less likely to play on with symptoms post e-learning. One academy youth player received *‘a leaflet and a chat with the medics’* and described how the information didn’t ‘*stick with him massively*,’ and that *‘it* (concussion) *wasn’t made a big deal*. *I wanted to highlight that*, *to get it across* (in the interview). *It wasn’t enough*.*’*

In the amateur game, concussion knowledge was seemingly obtained serendipitously, rather than information being delivered in a targeted way. Some concussion information had been highlighted through films, posters, or media stories: *‘people saw the story about the 19-year-old who got concussed and sadly passed away in her sleep*. *That hit home*. *It’s not something to be shrugged off*.*’* However, concussion misinformation was also spread through the media. M17 recalled seeing a televised professional match where a player was *‘clearly knocked out’* but *‘put back* [into play] *straightaway*’. He described feeling ‘*really conflicted’* because he *‘thought at every level it was meant to be two or three weeks off*.*’* M17 then indicated the regularity of similar events: *‘there’s so many ones where you see it and they’re back on within a week*.*’* M14 also felt that the glorification of excessive contact in the media was dangerous and could increase the risk of concussion and inappropriate RTP practices (Table 2, Quote 6).

For other amateur participants, concussion had only been discussed in passing, for example, being told prior to kick-off *‘if you have a head knock just come off*, *don’t be a hero’* (W6, M8). One grassroots team had a compulsory education session, but as a one-off that was never revisited despite the *‘high turnover of players’* within the team (W3). Other participants had misconceptions about concussion presentation: *‘I didn’t have all of the symptoms of concussion*, *so I didn’t think it would count’*, *‘anything less than a clear knockout was not treated as concussion’*. Other misconceptions were that concussion was a *‘one-day thing*,*’ ‘you’d wake up the next morning and be fine’*, and M12 recalled an attitude amongst his team where the need for medical support was considered binary: *‘you either went to hospital or played on’*. M17 had come to understand that concussion was a *‘scale’* rather than a binary *‘yes or no’* but did express how the complexity of the injury meant that this level of understanding took considerable time to achieve.

### Recovering from concussion

#### Availability of support throughout recovery

Participants who experienced a concussion received practical and emotional support from coaches, parents and teammates. At the amateur level, support included food shopping, offering transport to medical appointments, and checking in with concussed players. Players valued this support from coaches, such as checking in and assisting with the RTP process: *‘it gave you hope that you were gonna get back on the pitch’* (W3, M8), particularly when players were at a crucial stage in their rugby career, such as moving up a league. Participants appreciated explicit guidance encouraging them to take time off, such as: *‘you’ve had a concussion*, *don’t worry about training next week’* (M11, M8), but were irritated when coaches did not acknowledge concussions reported to them (Table 3, Quote 1). Injury visibility was highlighted as a factor that influenced support during recovery: *‘I guess they saw me coming to college*, *and I didn’t look injured*. *They probably didn’t think anything of it’* (M9), *‘In general*, *people are really good about my leg*, *because my leg you can see*. *It’s different with your brain*, *people don’t know you’re having a bad day*, *it’s not visible so it’s a bit harder’* (W5).

**Table 3:** Supporting quotes for ’Recovering from concussion’.

*Quote*	*Code*	
1	W1	I was quite upset at the fact that when I sent an email to the coaches at camp saying I was concussed I didn’t get any response from them. That was quite rubbish. I felt like I’d ruined my chances to play at that level. There was no follow-up after that date, even though I was supposed to be back in camp.
2	W10	Because I didn’t know I spent ages resting and symptoms weren’t going anywhere. It wasn’t until six months later that I started getting neck treatment and that’s really given me relief. I guess I’d like more information on what the complications could be.
3	M17	People need to discuss it and talk about their own experiences for other people to understand if they’ve had it or not. People will start talking about it in a way where it isn’t a taboo subject.
4	W15	One girl on my team got a real bad concussion. No-one ever heard from her again, and she was our social secretary. All of a sudden, she wouldn’t talk to anyone, wouldn’t do anything. I think she probably could have done with more support from the club.
5	M14	Because of the military I get paid whether I’m injured or not. I want to talk to the parents about this, at 16, 17 years, going into work why he needs insurance. If he’s injured, how is he going to work, how is he going to survive?
6	M11	The contracts say they’ll get £100,000 per year, but they get £15,000 and when they do a certain number of appearances that year, they get the other £80,000 after that. Clubs are very good at not playing players so they don’t get their appearance bonus.
7	M11	He said he absolutely had to fight to get any money out of his contact to help him with his recovery. He said he couldn’t work for six months, and the club was not giving him any care. He was sending emails and emails, and nothing happened. He had to fight to get a slither of what his contract should have been.
8	W19	Looking back, it was fantastic. After the return to contact block the coaches would ask how you were. If people didn’t feel good, they would go back to the phase before. It was really good because I didn’t have to go straight into training and bash someone. These programmes not only break the stigma but help you return confidently.
9	M11	We had to come at lunchtime and hit some tackle bags to do the contact element of the training instead of waiting for the following Monday. They said come to the contact session so you can play Saturday instead of waiting for sessions. There wasn’t a, you can come back in your own time.

Women participants alluded to there being gender bias in the treatment of women with injuries, specifically expressing that women and girls were less likely to have their symptoms taken seriously than men and boys. This sexism was reportedly present despite players and coaches having a positive working relationship: *‘he cares about us*. *But he’s a guy*, *he doesn’t necessarily see an injury for what it is’* (W3). This gender bias stemmed from men and women: *‘I think response-to-injury is gendered*, *like girls don’t take other girls seriously’* (W19).

Participants often struggled to access further information even when they sought medical attention for their concussion: *‘I got a little bit of information from the doctor*, *but to be honest nothing*. *I’ve had to do my own research’* (W10, W6). W10 recalled how she suffered a concussion, but because she did not know how the neck could be injured during a concussive event, experienced an extended recovery (Table 3, Quote 2). Participants expressed a desire to further their understanding of recovery processes to improve their well-being and suggested that mandatory union-provided education would be beneficial, noting that there were pre-existing opportunities to provide education, such as pre-season or pathway introduction meetings. Furthermore, group discussion was perceived to be highly important for breaking any concussion stigma (Table 3, Quote 3).

A need for greater social support post-concussion was highlighted by participants. Concussed persons were reportedly at risk of becoming isolated as people *‘naturally drifted away’* from the team (W15). It was suggested that these feelings of isolation could be prevented if teams were able to offer more support (Table 3, Quote 4). To maintain some involvement with the team, participants reported attending training but not taking part: *‘I couldn’t join in with the contact*, *but I would run*. *I just wanted to be there’* (W5). Other participants would want to get involved with coaching: *‘I’d definitely go and help them with forward drills’* (W3) or join the committee to have an admin role within the team.

Participants also expressed the importance of having financial support during recovery but noted that being insured to play rugby was not typical, particularly at the lower levels (Table 3, Quote 5). Financial strain experienced during medical leave from work could lead players to RTP earlier than was medically advised, particularly for professional players where income could be performance dependent (Table 3, Quote 6). It was proposed that professional players should have a clause in their contracts whereby *‘if you retire from injury*, *the union demands that you are paid like a minimum of 80% of whatever was left in your contract’* (M11). M11 recalled a story of a colleague who had played professionally but struggled to receive medical support from the club when his concussion led him to leave rugby (Table 3, Quote 7).

#### Experiences of return to play programmes

Only participants who had been concussed within high-performance university teams or at the elite level reported following a structured RTP programme. One high-performance participant (W19) had been associated with a research project investigating RTP and praised the approach of coaches and researchers working closely together to support concussed players. W19 described involvement within this project as having a *‘proper RTP programme*’ with progressive exercise supervised by the researcher, access to brochures, and to a specific strength and conditioning programme for concussed players. This structured approach was valuable in supporting the mental health of concussed players: *‘it was so genuinely useful in terms of your mental health and well-being*, *they were checking on me everyday*. *I felt really important*. *That prepped me so well*.*’* Once medically cleared, players were supported by the coach who facilitated return-to-contact blocks (Table 3, Quote 8).

This comprehensive concussion management approach encouraged an attitude change amongst the team, whereby concussions were respected and responded to proactively. W19 recalled an event where a concussed player went to the gym to train and was *‘removed by the coach and not allowed to train the following week either*. *The coach did it to make the point that she shouldn’t be training*.*’* The reporting of this player’s behaviour was encouraged by the coach and *‘created a culture where people were keeping tabs on who was concussed’* and acting in their best interest. W19 recalled that those who did not follow the RTP programme *‘had nothing to help them build back up*’, lacked confidence and *‘just weren’t ready’*. She described a *‘massive problem’* where people were returning *‘straight to tackling’* without opportunities to gradually increase the level of contact.

Such positive experiences of RTP programmes were not shared by other participants, particularly those in high-performance teams (W15, M11). These programmes were technically adhered to but served to *‘tick a box’* rather than support a player during return to competition. M11 recalled how coaches would enforce attendance at additional training sessions to complete RTP protocols as quickly as possible (Table 3, Quote 9) and that players experiencing prolonged recovery were *‘definitely pressured’* to return as soon as possible. It was suggested that those who saw rugby as a potential career were more vulnerable to having their RTP times shortened by this pressure, but M11 knew rugby was not his *‘be all and end all’* thus *‘felt more comfortable’* resisting the coaches wishes: *‘I had to be like*, *no*, *I am still at this stage*. *They were a bit short with me*. *It was never formally required that you were back for a certain time*, *but you felt the pressure’*. Similarly, W15 noted a need to be comfortable resisting coach pressure: *‘you need to be strong enough to dig your heels in and say actually no*, *I’m not fit’*.

Despite having been pressured to RTP, M11 sympathised with the coaches *‘to an extent’* as they *‘also had a need to perform’* and that he could *‘completely understand from their point of view’* in situations where specialised players (scrum half or fly half) were unable to play due to concussion. High match volume further increased this pressure to return: *‘we were in BUCS* (British Universities and Colleges Sport) *Super Rugby on the Wednesday and at risk of being relegated from the championship league on a Sunday*, *we had to have players to field for both matches*.*’*

### Readiness to return to play

Participants reported psychological changes post-concussion, such as a loss of confidence (W19, W5). W19 gave a detailed account of the psychological changes she experienced following a childhood concussion: *‘It was a case of two weeks off then back into it*. *But the joy I had for it had gone completely*, *I loved tackling but now I was afraid of it*.*’* She explained how she took pride from being known within the team as a good tackler, so alongside the loss of confidence she *‘basically lost* [her] *identity*.*’* She recalled how the *‘coaches were completely unaware’* and that she was *‘really ashamed to tell* [her] *mum*.*’* This participant ended up leaving her club ‘*without saying goodbye’* to her friends: *‘I’d rather say to people I just have to go than admit I’ve got really bad anxiety*.*’* This post-concussion loss of confidence was shared by W5 as she returned to match play: *‘I was put on the bench a couple of times that season but I didn’t play*. *I didn’t want to*, *I was scared*.*’* She recalls that when she did return to match-play for the first time, she *‘was rubbish’* and *‘didn’t want the ball*.*’* Having realised that she no longer enjoyed playing, she considered that day her ‘*last match’* and retired for a couple of years.

## Discussion

In this study, rugby players were interviewed to explore their experiences of concussion at a range of playing levels. Concussion experiences across the sport were highly variable in accordance with the social environment of the team. The appropriateness of the immediate concussion response was influenced by symptom visibility, potential implications for the ability of the team to field enough players, and a poor understanding of the injury. During the recovery period, participants were generally unsure of how to support others during RTP and highlighted the loss of confidence experienced, by themselves and their peers, post-concussion. These findings highlight the need to improve the accessibility of concussion support and players’ and coaches’ knowledge of concussion recovery.

Consistent with previous research [19, 26], the findings of the current study suggest that the stigma of ’being soft’ and a lack of concussion knowledge can impede removal from play decisions and healthcare-seeking behaviours. Despite acknowledging the importance of rest and removal from play post-concussion, pressure from the team could result in knowingly unsafe concussion practices. For example, one participant’s team had knowingly fielded a concussed player to avoid financial penalties from the league. Whilst regretting this decision, the participant expressed that the financial vulnerability of the club meant that the penalty would have significant implications for the future of her team. Alteration of the penalty format to remove financial punishment may prevent similar instances from happening in the future, however, there remains the potential that teams may take advantage of such rulings to gain a winning advantage. A solution to such an issue is therefore challenging but warrants further consideration given its potential implications for the current and future health and well-being of players.

Factors influencing the concussion response reported in this study concur with the literature, including perceived lack of severity [27], being ‘headstrong’ [28] and pressure to perform [29]. The gendered response to injury in the current study was unexpected and differed to literature relating to musculoskeletal injury [30]. Indeed, in vignettes of student athletes that depicted an anterior cruciate ligament injury, no significant differences between patient gender were found between the treatment compliance expectations, oversight expectations, and social support offered by the medical staff [30]. However, concussion may be more vulnerable to gendered treatment, given that diagnosis of concussion commonly relies on self-reported symptoms [31] and may rather be in accord with findings with conditions such as chronic pain, in which gender has been reported to influence treatment approaches, potentially due to the presence of pain in the absence of objective or detectable symptoms [32]. Nonetheless, it is pertinent to note that there is a dearth of research and further studies are warranted to investigate the extent to which such bias may exist in rugby.

This study highlighted how media coverage of concussion and rugby may influence players’ attitudes and responses to such injury. Media coverage of concussion raised awareness of the potential severity of the injury, but participants felt misled when incorrect concussion procedures were televised without acknowledgement of such errors. Additionally, the dangers of promoting excessively forceful tackling in social media content were highlighted by participants and could lead to youth players copying such behaviours. Similar inappropriate framing of sports injuries has been exemplified in the textual analysis of media reporting of limb injuries in American football players [33]. Specifically, in case studies of two athletes with bodily injuries, 44–54% of media coverage was negative, down-playing injury severity, normalising playing through injury, and stigmatising removal from play [33]. Indeed, the consumption of risk-glorifying media can influence risk-taking behaviour and the social norms surrounding risk, causing habituation of risky behaviours [34]. As participants suggested that adolescents may be more vulnerable to the stigma of concussion reporting and engage in performative bravery, speculatively, such videos could exacerbate this vulnerability through the normalisation and praise of excessively forceful contact. There is a need to exemplify optimal care surrounding concussions in the media. For example, at televised matches, there is an opportunity to further educate the public and promote positive concussion behaviours.

Knowledge gaps were evident in terms of how recovery could be supported and how best +to navigate RTP. Knowledge gaps may result from the assumption that players will seek post-concussion medical guidance as recommended [18] and that doctors could communicate this advice, thereby allowing greater focus in education programmes on the immediate response to concussion. Whilst this may be a reasonable assumption in theory, players [17], and the general public [35] do not typically seek healthcare post-concussion. Therefore, medical staff cannot be exclusively relied upon for the dissemination of current recovery guidelines, and consequently, there is a need to improve stakeholder understanding of supporting recovery from concussion.

Resolution of concussion symptoms is considered a milestone in the recovery process and allows for RTP protocols to be commenced. However, in accord with Choudhury et al [20], participants in the current study indicated that post-concussion loss of confidence existed long after the resolution of symptoms. This loss of confidence may result from a fear of recurrent concussion, fear of returning to sport, and fear of losing playing status [36]. Although rugby-specific RTP programmes detail a stepped approach, with the restoration of confidence identified as a goal, little advice to support psychological or social recovery is offered [18, 21, 37]. Indeed, the current post-symptom resolution RTP programme developed by World Rugby encompasses six stages (i) initial rest; ii) light aerobic exercise; iii) sport-specific exercise; iv) non-contact training drills; v) full contact practice; and vi) return to sport) but the jump from non-contact to full-contact training may be perceived as too rapid a progression for those new to rugby, or those experiencing a loss of confidence. Whilst players should progress through the stages at their own pace, the pressure to RTP early and perception that completion of an RTP programme was a ‘tick-box’ exercise (M11) may preclude this gradual progression. Less confident or inexperienced players may therefore benefit from an additional stage between step four and five of the RTP programme, whereby contact using tackle bags only is re-introduced as a stand-alone session. Coach education programmes may also benefit from information surrounding potential psycho-social changes post-concussion, or how to rebuild the confidence of returning players.

This study offers valuable insights regarding how players experience and recover from concussions in rugby. However, it is imperative that certain limitations are acknowledged. Whilst all participants that volunteered were offered an interview, players that did not wish to participate in the interview may have offered differing insights to those that completed the interviews. An online interview format was chosen to prevent location from restricting participant engagement. However, the ethnicity of participants or socio-economic status was not disclosed, therefore the extent to which the participant pool is representative of the ethnicities and socio-economic profiles of those that play rugby cannot be ascertained. As such, future research would benefit from the use of purposive sampling to reflect the wider rugby playing population. The results are derived on the basis of all who participated but certain participant’s quotes were utilised more as they provided more eloquent examples to illustrate participant experiences. Where a participant highlighted a unique event, their experience was not considered less valuable because their experience does not align with the norm, rather they were considered a voice for their club or team and were not undervalued.

Participants spoke powerfully about the negative experiences they experienced post-concussion, including difficulty accessing support during recovery, loss of confidence and the ‘stigma of being soft’. In order to protect player welfare, such barriers to optimal recovery must be further understood, and players supported better in terms of access to medical care, the social environment of the team, and dissemination of further information about recovery processes. In the future, a supporting study that uses a survey methodology may be beneficial to understand the positions of a larger population.

## Supporting information

S1 File Interview guide. (DOCX)
