# Supplementary material for: Concussion response and recovery in men and women’s rugby union: A reflexive thematic analysis of player interviews
Source: PLoS One. 2024 Apr 30;19(4):e0296646. doi: 10.1371/journal.pone.0296646 (PMC11060544; doi:10.1371/journal.pone.0296646)
Supplement: S1 File — (DOCX) [file pone.0296646.s001.docx]

# Interview guide

1. Participant consent will be obtained prior to the start of the interview, and the interviewer will introduce themselves and explain the purpose of the interview.

*We are conducting these interviews to explore your experiences of rugby and how that may, or may not relate to your gender. Just a reminder that anything you say will be anonymised in any publications, and you are more than welcome to stop the interview at any time.*

1. The interview then begins by asking the participants:

*-Please could you tell me about your journey into rugby?*

The interviewer will take notes on demographics or specific experiences that may be relevant for probing questions later in the interview.

1. How did you get involved in rugby, what made you want to start playing rugby
2. Did you play any other sports before playing rugby
3. What resources do you have available to you to help improve your performance
4. How valuable to you find these / would you like access to these resources & what do you think is stopping you from getting access?

- Coaching, a place to practice/changing rooms/ s&c / working equipment/ scrum machines

1. What resources do you have available to you to help improve your safety
2. How valuable to you find these / would you like access to these resources & what do you think is stopping you from getting access?
3. If they’ve played rugby for a long time, how did these resources differ by level/ through the age grades
4. How do you think things would change if you had access to more healthcare resources? What resources would you like?
5. (if applicable) When you approach any medical staff with an injury whilst playing rugby, do you feel you are taken seriously?
6. What teams do your club currently have, what’s stopping expansion? How easy is it to access this club?
7. Are resources shared equally within this club?
8. What else should I know about this area to investigate this well?
